# Effectiveness of a Health Education Program to Reduce Recurrence of Stroke by Controlling Modifiable Risk Factors in a Specialized Hospital in Bangladesh: Randomized Controlled Trial

**DOI:** 10.2196/72233

**Published:** 2025-05-27

**Authors:** Mahabuba Afrin, K A T M Ehsanul Huq, Sharif Uddin Khan, Subir Chandra Das, Mohammad Shah Jahirul Hoque Chowdhury, Yasuko Fukuoka, Yasuko Fukushima, Michiko Moriyama

**Affiliations:** 1 Graduate School of Biomedical and Health Sciences Hiroshima University Hiroshima Japan; 2 National Institute of Neurosciences and Hospital Dhaka Bangladesh; 3 Department of Nursing Ube Frontier University Yamaguchi Japan

**Keywords:** stroke, health education, recurrence, modifiable risk factors

## Abstract

**Background:**

Health education could be an effective way to increase knowledge regarding behavioral changes to prevent the recurrence of stroke; however, the evidence is ambiguous. A lack of both knowledge and compliance with treatment to control modifiable risk factors and unhealthy lifestyles increases the risk of stroke recurrence.

**Objective:**

This study aimed to evaluate the effectiveness of a health education program among patients with stroke postdischarge and their family caregivers in a tertiary specialized hospital in Bangladesh to reduce stroke recurrence by controlling modifiable risk factors.

**Methods:**

A parallel (1:1), open-label, prospective randomized controlled trial was conducted in Bangladesh. A total of 432 patients with first-time stroke, aged ≥18 years and a modified Rankin Scale (mRS) score of 0-4, were randomly enrolled at the National Institute of Neuroscience & Hospital. We stratified the patients by age and type of stroke and randomly allocated to an intervention group (IG) and a control group (CG). We collected sociodemographic and clinical data by using a structured questionnaire. The IG received (self) management education, including monitoring blood pressure (BP), medication, diet, and exercise for 12 months, and the CG received usual care. The outcomes were (1) recurrence after 28 days of stroke and (2) all adverse events related to stroke after 12-month follow-up.

**Results:**

Of 432 patients (n=216, 50%, in each group), stroke recurrence was observed 14 (6.5%) patients in the IG and 8 (3.7%) patients in the CG, and the difference was not significant (*P*=.19). Death was lower in the IG (n=39, 18.1%, vs n=56, 25.9%) compared to the CG. In Cox regression analysis, the unadjusted model showed death was significant (hazard ratio [HR] 1.531, 95% CI 1.017-2.304; *P*=.04); however, the difference was not significant after adjusting the mRS score at baseline (HR 0.818, 95% CI 0.540-1.238; *P*=.34). Patients’ medication adherence significantly improved at 6-month (*P*<.001) and 12-month (*P*=.002) follow-up after the intervention.

**Conclusions:**

This study failed to demonstrate the effectiveness of health education in reducing recurrence, death, and stroke-related adverse events. However, health education enhanced medication adherence. Some causes of death could not be diagnosed due to inadequate health care systems. Further research with diagnostic facilities and a long observation period is necessary to explore the underlying cause of recurrence. The results suggest the importance of structuring acute care management for patients with stroke into the health care system of Bangladesh.

**Trial Registration:**

ClinicalTrials.gov NCT05520034; https://clinicaltrials.gov/ct2/show/NCT05520034

## Introduction

The stroke burden is a significant and growing public health concern. Stroke was the second-most fatal and third-most leading cause of disability worldwide in 2022. Its prevalence is rising rapidly in low- and middle-income countries (LMICs) [1]. Every year, more than 13.7 million strokes occur worldwide, which leads to 5.5 million deaths. Although strokes are more likely to affect older adults, there is an increased frequency of strokes among younger people [2,3]. In 2019, the foremost risk factors for stroke were elevated systolic blood pressure (BP), a high BMI, elevated fasting plasma glucose concentrations, and smoking [4]. Studies have indicated that the risk of recurrent stroke is the highest within the first few months after the initial event, and up to 25% of survivors experience a recurrent stroke within 5 years [5]. Recurrent strokes are associated with fatal outcomes, increased disability, and higher mortality rates compared to first-time strokes. It is essential to manage modifiable risk factors for reducing stroke recurrence and improving long-term outcomes for survivors of stroke [5].

In Bangladesh, stroke was the top cause of death per 100,000 population for both males and females and all ages in 2019 [6]. In 2022, Bangladesh had a stroke rate of 79.9%, 15.7%, and 4.6%, which was caused by strokes that involved ischemia, hemorrhage, and subarachnoid hemorrhage, respectively, and affected 1,139,000 people [7]. One study conducted in a tertiary medical university hospital in Bangladesh evaluated the rate of recurrence of stroke and found that the cumulative recurrence rate was 14.7% at 3 months, 15.3% at 6 months, 17.3% at 9 months, and 20% at 1 year. The most frequent age group was >75 years, which represented 44.4% of recurrent stroke cases [8].

Despite the advancement of thrombolytic therapy and acute stroke management, preventing the recurrence of stroke remains a significant challenge. Moreover, the ability of patients to manage complications and avoid underlying diseases, such as hypertension, diabetes, and dyslipidemia, is impacted by substandard primary care and a lack of referral mechanisms. Patients in underdeveloped health care systems are not consistently diagnosed or treated because of a lack of information, infrastructure, resources, shortage of equipment, a lack of diagnostic accuracy, a lack of health education, and budgetary constraints, which prevents them from undergoing periodical medical examinations [9,10].

One systematic review concluded that health education may improve BP targets but not improve other risk factors or reduce recurrent events, and patient education alone might not prevent the risk of recurrent stroke [11]. A study in Bangladesh found that a small sample size of 150 is insufficient for identifying the stroke recurrence frequency. A larger sample size and health education efforts are recommended to reduce recurrence [8]. Another study involving a sample size of 321 in Japan provided self-management health education for 6- and 24-month follow-up. This study reduced the recurrence rate by half after the intervention; however, the difference was not statistically significant for the prevention of stroke recurrence [12].

Since there is no established patient follow-up system and health education offered across hospitals in many high-income countries, this intervention study was conducted to incorporate patient health education following discharge from acute care hospitals. Even though hospitals provide health education at the time of discharge to improve patients’ self-motivation at home, patients with stroke are found to lack the knowledge to follow medical advice [13]. As most of the patients are taken care of by family caregivers, their health education can significantly reduce risk factors and complications related to stroke [14].

In Bangladesh, health education for patients with stroke regarding self-management skills at home from the hospital level is limited. The reasons could be sociocultural, economic, and political, as well as poor planning and improper implementation of health policies and programs within the health system. Patients with stroke and their family caregivers should know the importance of controlling modifiable risk factors [8]. Therefore, it is crucial to establish a well-organized patient–disease management education system.

Based on these findings, we hypothesized that a health education program among patients with first-time stroke and their family caregivers could reduce stroke recurrence by controlling modifiable risk factors. We tried to evaluate the effectiveness of a health education program in improving risk factors and reducing the recurrence of stroke and other stroke-related adverse events.

## Methods

### Study Design and Participants

This was a parallel (1:1), open-label, prospective randomized controlled trial and adhered to the CONSORT (Consolidated Standards of Reporting Trials) guidelines (Multimedia Appendix 1) [15]. This study was conducted at the National Institute of Neuroscience & Hospital (NINS&H) in Dhaka, Bangladesh, between October 2022 and March 2024. The NINS&H is the only state-run referral tertiary care hospital in Bangladesh focused on neurological disorders. Patients with neurological disorders, including strokes, and from different socioreligious groups visit this hospital from all over the country. We enrolled participants who had their first stroke and were in the inpatient department or the emergency department before discharge or who were visiting the outpatient department of the NINS&H. The participants were aged 18 years and above, both men and women, and were willing to participate in this study. We included patients with ischemic (large artery atherosclerosis, cardioembolism, small vessel occlusion [SVO], lacunar subtype, and transient ischemic attack [TIA]) strokes diagnosed by physicians. In addition, as a sufficient number of patients with hemorrhagic stroke visited the hospital, we included those patients too in order to obtain the required sample size. Enrolling both types of patients in this study helped us understand their clinical presentations, complications, and recovery patterns and also provided comprehensive data for better stroke management strategies and resource allocation in resource-limited settings. We included only patients with a modified Rankin Scale (mRS) score of 0-4. As most of the patients with stroke with mRS score=0-4 in Bangladesh have family caregivers to provide health care support, family caregivers, living with the patients and providing support to them, were also included [16]. We excluded patients who had mRS scores of >4, had mental illness or cognitive impairment (diagnosed cases), had participated in other clinical trials, and had multiorgan failure or terminal disease.

### Randomization

Participants were randomly assigned to an intervention group (IG) and a control group (CG) in a 1:1 ratio using a pregenerated allocation list based on block randomization with variable block sizes (2-4). The list was stratified by age group (18-64 vs ≥65 years) and stroke type (1: large artery atherosclerosis; 2: cardioembolism; 3: SVO/lacunar/TIA; and 4: hemorrhage). Statistics for the age of patients with recurrent stroke and the types of strokes were not available at the study site, so we used the study findings as references [17,18]. We used this stratified block randomization technique as recurrence rates varied depending on the age and type of the stroke [19]. This randomization procedure was conducted by an experienced investigator who was not involved in the study intervention or analysis. This design minimized bias by controlling key confounders at this stage.

### Sampling Technique and Study Procedures

A convenient sampling technique was used to enroll participants. Neurologists at the hospital and research assistant (RA) nurses identified patients with stroke, then checked the patients’ eligibility criteria by referring to their medical records. We used sealed envelopes to keep the serial number of the allocated randomization group. These envelopes were prepared and opened sequentially only after participant enrollment to allocate patients to the respective groups. The RA nurses obtained each participant’s sociodemographic information (age, gender, marital status, education, occupation, living status) and clinical data (medical history, comorbidities, treatments, prescribed medications, self-reported medication adherence, types of stroke, mRS score, and risk factors of stroke). Additionally, laboratory data, including glycated hemoglobin (HbA1c), lipid profile, and BP, were obtained by interviewing the participants and from hospital medical records. The medication adherence questionnaire included participants’ responses to whether they followed the doctor’s prescription for taking medications.

### Intervention Group

**Figure 1:**
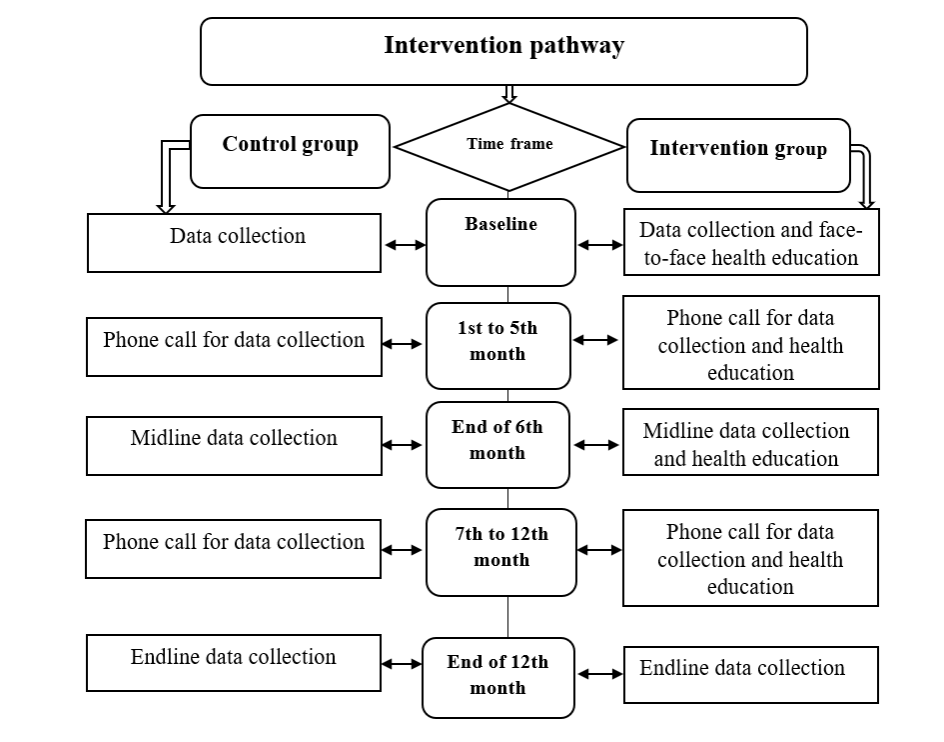
Study activities of the IG (n=216) and the CG (n=216). CG: control group; IG: intervention group.

After enrollment at the hospital, RA nurses conducted a face-to-face health education and video presentation for the patients and their family caregivers for 45 minutes. The RA nurses followed up with the participants through phone calls every month (months 1-3, twice a month; months 4-6, once a month) and sent SMS text messages to remind them to record their daily monitoring in a health education book. At midline after 6 months and at endline after 12 months, the RA nurses called the participants to the hospital for follow-up visits, provided the same health education as baseline, collected data, and conducted laboratory tests. Figure 1 shows the intervention pathway.

If a patient and their family caregiver were unable to visit the hospital for any reason, then the RA nurses provided health education over the telephone and advised them to undergo the laboratory test at a nearby hospital. The RA nurses also visited the participants’ homes to provide health education and for sample collection, if needed. If an RA nurse failed to communicate with a patient/caregiver over a phone call for 3 consecutive months, the patient/caregiver was considered a dropout. For participants, 6 months of follow-up visits at the hospital were found to be low due to economic, social, and transportation difficulties, particularly in rural areas. The RA nurses arranged home visits at 12-month follow-up. This significantly increased participant involvement at 12 months compared to 6-month follow-up visits, emphasizing the importance of flexible research designs in LMICs, such as Bangladesh. This study has been described elsewhere [20].

#### Health Education Procedures

Trained RA nurses provided health education sessions to participants using a researcher-developed booklet, which was provided to the patients for self-education at home. We delivered 2 health education sessions using a multimedia approach, which included visual aids and interactive discussions, alongside the booklet material. For patients who could not attend intervention sessions, health education was provided at their bedside using the same educational booklet to maintain consistency and ensure accessibility of information.

#### Components of the Health Education Intervention

##### Lecture-Based Education

A booklet-based health education session was conducted for about 20 minutes. Each participant received 2 sessions of health education: once at enrollment and again during the 6-month follow-up hospital visit. The booklet guided patients on how to interpret food labels to manage stroke-related risk factors and included a reminder section for upcoming clinic appointments. Additionally, it featured a health-monitoring notebook for following daily BP, physical activity, and medication adherence by patients or caregivers.

##### Video-Based Education

Subsequently, participants watched a 15-minute educational video covering stroke risk factors, their management, and their prevention strategies. The educational video included simulated demonstrations of essential tasks, such as measuring the BP, organizing medications using boxes, and using salt-measuring spoons.

Following this, a physiotherapist conducted a 10-minute orientation session to teach participants about appropriate home-based daily physical exercises. To reinforce learning and support retention, RA nurses and physicians repeated the educational content during the 6-month follow-up visit according to the protocol.

Monthly motivational SMS reminders were sent to patients and caregivers to encourage adherence to daily BP monitoring, reduced salt intake, healthier eating habits, regular physical activity, and consistent medication use.

### Control Group

Participants received standard medical care as per the hospital’s protocols. As part of routine poststroke care, they received medical treatment, including prescribed medications and follow-up based on the attending physician’s recommendations. Importantly, no additional health education related to stroke recurrence prevention was provided during the intervention period. This ensured that only the IG was exposed to the educational component of the study.

### Sample Size

We considered the stroke recurrence rate to be 20% [8]. The incidence rate of recurrence was 1.9/100 and 3.8/100 person-years for the IG and the CG, respectively, with 50% reduction [12] and 80% power. The expected proportion of outcomes from the IG was 10%. Here, “n” was the sample size, according to assumptions P_1_ was the proportion of outcomes from the IG (10%), P_2_ was the proportion of outcomes from the CG (20%), α was the level of significance (5%), 1 – β was the power of the test (80%), Z_1–α/2_=Z α value level of significance (1.96), and Z_1–β_=Z β value level of the corresponding level of power (0.84). The estimated sample size was 196 for each arm (total 196 × 2=392 for both arms). Considering a 10% dropout rate, the final sample size was 432 (n=216, 50%, for each arm).

### Operational Definitions

#### Recurrence of Stroke

Recurrence was defined as a new stroke event occurring at least 28 days after the incident event [17] confirmed by a physician who treated and diagnosed the patients in this study. We considered this definition of recurrence as >28 days to see the effects of the intervention in reducing stroke recurrence.

#### Adverse Events

Adverse events, including all causes of death and cardiovascular events, referred to any case that required a diagnosis. For instance, the physician treating and diagnosing the participants confirmed whether any recurrence of stroke, new onset of acute myocardial infarction and unstable angina, heart failure, and peripheral arterial disease required hospitalization.

#### Outcomes

The primary outcome of this study was to reduce the recurrence rate of stroke by providing health education after 12 months of follow-up. The secondary outcomes were to (1) reduce all adverse events (stroke recurrence, all causes of death, and cardiovascular events) and (2) increase medication adherence by providing health education after a 12-month follow-up period.

The effectiveness of health education was evaluated using 2 structured tools: a knowledge assessment questionnaire focused on stroke risk factors, symptoms, and prevention strategies and a behavior change questionnaire designed to measure lifestyle modifications, such as diet, physical activity, medication adherence, and BP monitoring. All tools were developed by the research team. These results will be published in a separate paper.

### Data Monitoring and Quality Assurance

We conducted pretesting to observe the RA nurses’ consent-taking, data collection, and physical examination procedures using 5% of the estimated samples of the total 432 participants and changed the procedures where necessary. These participants were not included in the main study. In our study, investigators (medical professionals) had direct involvement in patient care. They held regular biweekly meetings with the RA nurses to discuss the progress of the study and resolve any confusion and concerns. Furthermore, as part of routine quality control checking, the investigators performed field testing by independently verifying 5% of the data collected from participants on the same day. The RA nurses consulted with the investigators for any queries: questionnaires were completed by the RA nurses, who then input the information into a password-protected computer to maintain confidentiality. The investigators examined the information and answered the questions.

### Statistical Analysis

An intention-to-treat (ITT) analysis was conducted. Descriptive statistics were performed to assess baseline data. Categorical variables were reported as frequencies and percentages and compared between the IG and the CG using the chi-square (*χ*^2^) test or the Fisher exact test, where applicable. Continuous variables were reported as the mean (SD), and after checking the normality test, we performed the Mann-Whitney U test as our variables were not normally distributed. To estimate the time to events during the 12-month follow-up period from enrollment to the occurrence of events, study completion, dropout, and death, we used Kaplan-Meier estimates and compared them between the IG and the CG using the log-rank test. To compare the primary and secondary endpoints between the IG and the CG, we used Cox proportional hazards regression, and results were presented as hazard ratios (HRs) with 95% CIs. Because statistical significance was observed in mRS scores at baseline, we adjusted for the mRS score as a covariate in the Cox regression model to mitigate its potential impact on the primary and secondary outcomes. We also conducted the *χ*^2^ test for medication adherence to compare the 2 groups. The significance level was set as *P*<.05. Data were analyzed using SPSS version 27.0 (IBM Corp).

### Ethical Considerations

This study was approved by the Ethical Review Committee of the NINS&H (approval number: IRB/NINS/2022/151), Dhaka, Bangladesh. All participants provided written informed consent. In case the patients could not take care of themselves and needed family caregivers’ support for their daily activities, we obtained written informed consent from the family caregivers instead, considering them as participants. Data were deidentified and assigned unique identification numbers for analysis, and no personal information was used in any publication or presentation resulting from this research. Allocation concealment was securely maintained by the principal investigator (PI) in a password-protected personal computer. Participants were compensated when they performed laboratory tests by themselves, and we reimbursed the same amount based on the receipt. To maintain ethical standards, health education materials were distributed to all CG participants after the intervention period was completed.

## Results

### Participant Details

**Figure 2:**
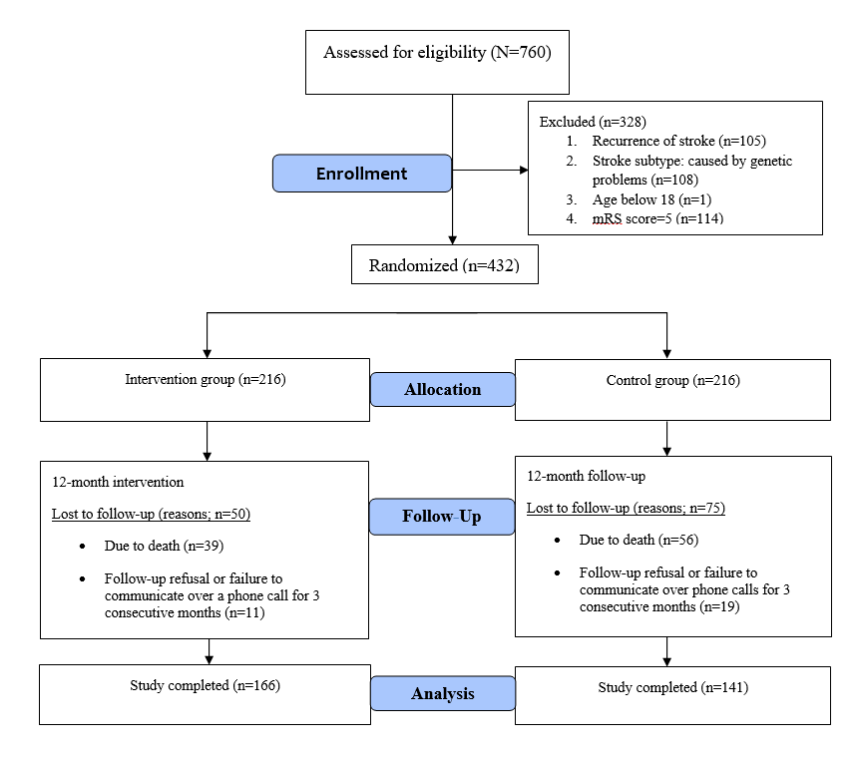
Study flowchart based on the CONSORT statement.

Of the 760 participants, we excluded 328 (43.2%) participants, and 432 (56.8%) met the inclusion criteria and consented to participate in the study. The study flowchart is presented in Figure 2. Subsequently, participants were equally allocated to the IG (n=216, 50%) and the CG (n=216, 50%). After 12 months of follow-up, 307 (71.1%) participants completed the study (IG: 166/216, 76.9 %; CG: 141/216, 65.3%) and 95 (22%) died (IG: 39/216, 18.1%; CG: 56/216, 25.9%). Furthermore, 30 (6.9%) participants dropped out (IG: 11/216, 5.1%; CG: 19/216, 8.8%).

### Sociodemographic Characteristics and Clinical Profiles of Participants

The average age of the participants was 55.2 years (SD 13.5), with 273 (63.2%) males and 263 (60.9%) participants from rural areas. The mean duration of onset of stroke to enrollment (*P*<.001), dyslipidemia (*P*=.03), taking hyperlipidemic medication (*P*=.05), and mRS score=1-3 (*P*<.001) were significantly higher in the IG compared to the CG. In contrast, the CG had significantly more rural residents (*P*=.02), a history of myocardial infarction (*P*=.01), and mRS score=4 (*P*<.001). Age (*P*=.19) and stroke types (*P*=.96) were well allocated (Table 1).

**Table 1:** Comparison of baseline characteristics and clinical profiles between the IG^a^ and the CG^b^.

Characteristics			Total participants (N=432)		IG (n=216)		CG (n=216)		*P* value
	Age (years), mean (SD)	55.2 (13.5)		54.4 (13.7)		56.1 (13.2)		.19^c^	
	Males, n (%)	273 (63.2)		137 (63.4)		136 (63.0)		.92^d^	
	Married, n (%)	427 (98.8)		215 (99.5)		212 (98.1)		.18^d^	
	Living with family, n (%)	425 (98.4)		212 (98.1)		213 (98.6)		.70^d^	
	Education: literate, n (%)	309 (71.5)		160 (74.1)		149 (68.9)		.24^d^	
	Employed, n (%)	222 (51.4)		116 (53.7)		106 (49.1)		.34^d^	
	Residence: rural, n (%)	263 (60.9)		120 (55.6)		143 (66.2)		.02^d^	
	Monthly family income (US $)	184.39 (169.72)		189.45 (141.07)		174.05 (194.22)		.07^c^	
	Alcohol habit, n (%)	16 (3.7)		10 (4.6)		6 (1.4)		.31^e^	
	Smoking, n (%)	136 (31.5)		69 (31.9)		67 (31.0)		.84^d^	
**Clinical history of the patient**									
	Time from onset of stroke to enrollment (days), mean (SD)	7.75 (6.213)		8.85 (6.67)		6.64 (5.51)		<.001^f,g^	
	History of hypertension, n (%)	315 (72.9)		156 (72.2)		159 (73.6)		.75^d^	
	History of diabetes mellitus, n (%)	91 (21.1)		43 (19.9)		48 (22.2)		.56^d^	
	History of dyslipidemia, n (%)	295 (68.3)		158 (73.1)		137 (63.4)		.03^f^	
	History of arrhythmia, n (%)	30 (6.9)		15 (6.9)		15 (6.9)		.99^d^	
	History of myocardial infarction, n (%)	95 (22.0)		36 (16.7)		59 (27.3)		.01^d^^,f^	
**Stroke type, n (%)**									
	Cardioembolism	69 (16.0)		32 (14.8)		37 (17.1)		.96^e^	
	Atherothrombosis	131 (30.3)		65 (30.1)		66 (30.6)		.96^e^	
	Lacunar subtype	7 (1.6)		4 (1.9)		3 (1.4)		.96^e^	
	Hemorrhage	223 (51.6)		114 (52.3)		109 (50.5)		.96^e^	
	TIA^h^	2 (0.5)		1 (0.5)		1 (0.5)		.96^e^	
**Medications, n (%)**									
	Antihypertensives, n (%)	317 (73.4)		158 (73.1)		159 (73.6)		.91^d^	
	Antidiabetics, n (%)	92 (21.3)		43 (19.9)		49 (22.7)		.48^d^	
	Antihyperlipidemics	295 (68.3)		157 (72.7)		138 (63.9)		.05^d,f^	
	Antiarrhythmics	31 (7.2)		15 (6.9)		16 (7.4)		.85^d^	
	Cardiac drugs	95 (22.0)		36 (16.7)		59 (27.3)		.01^d^^,f^	
**mRS^i^ score, n (%)**									
	0	1 (0.2)		0		1 (0.5)		<.001^e,f^	
	1	57 (13.2)		48 (22.2)		9 (4.2)		<.001^e,f^	
	2	34 (7.9)		21 (9.7)		13 (6.0)		<.001^e,f^	
	3	152 (35.2)		84 (38.9)		68 (31.5)		<.001^e,f^	
	4	188 (43.5)		63 (29.2)		125 (57.9)		<.001^e,f^	

^a^IG: intervention group.

^b^CG: control group.

^c^Mann-Whitney U test.

^d^Chi-square test.

^e^Fisher exact test.

^f^*P*<.05.

^g^*t* Test.

^h^TIA: transient ischemic attack.

^i^mRS: modified Rankin Scale.

### Primary Endpoint

The recurrence rate after 28 days was 5.1% (22/432) in the total participants, 6.5% (14/216) in the IG, and 3.7% (8/216) in the CG, and the difference was not statistically significant (*P*=.19) between the groups (Table 2).

**Table 2:** Comparison of stroke recurrence between the IGa and the CGb during 12-month follow-up.

Recurrence onset	Total participants (N=432), n (%)	IG (n=216), n (%)	CG (n=216), n (%)	*χ*^2^ (*df*)	*P* value
Within 28 days	26 (6.0)	11 (5.1)	15 (6.9)	0.66 (1)	.42
After 28 days	22 (5.1)	14 (6.5)	8 (3.7)	1.73 (1)	.19
Total	48 (11.1)	25 (11.5)	23 (10.6)	0.09 (1)	.76

^a^IG: intervention group.

^b^CG: control group.

### Secondary Endpoints

**Figure 3:**
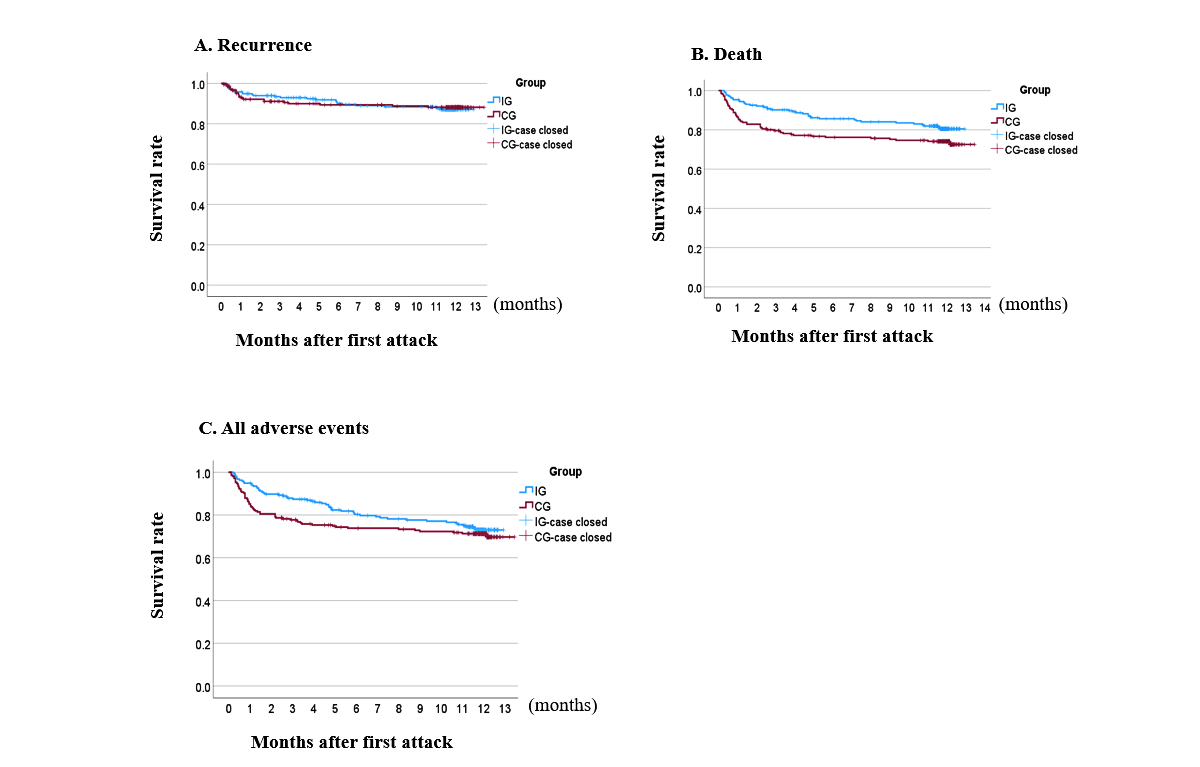
Kaplan-Meier survival curve estimates the survival rate (days) for 12-month follow-up from the onset of the first attack: (A) recurrence, (B) death, and (C) all adverse events (recurrence, death, and cardiac events). CG: control group; IG: intervention group.

The Kaplan-Meier survival curves in Figure 3A-3C show the estimated survival days for recurrence, death, and all adverse events at 12-month follow-up. These graphs illustrate time-to-event differences between the IG and the CG over the 12-month follow-up period.

### Stroke-Related Events

The HR for recurrence was 0.978 (95% CI 0.555-1.722, *P*=.94), and the total number of deaths was 95 (22%), with 39 (18.1%) deaths in the IG and 56 (25.9%) in the CG, and the difference was significant (HR 1.531, 95% CI 1.017-2.304; *P*=.04). The observed recurrence rate was significantly lower than expected, as mentioned before at 6.5% (14/216) in the IG and 3.7% (8/216) in the CG. The total number of all stroke-related adverse events, including recurrence, death, and cardiac events, in both groups was 116 (26.9%), with 54 (25%) adverse events in the IG and 62 (28.7%) in the CG, and the difference was not significant (HR 1.216, 95% CI 0.844-1.752; *P*=.29). To account for the significant baseline difference in mRS scores between the 2 groups, deaths were found to be not statistically significant after adjusting for the mRS score as a covariate. The adjusted HR was 1.234 (95% CI 0.693-2.197, *P*=.48) for recurrence, 0.818 (95% CI 0.540-1.238, *P*=.34) for death, and 1.016 (95% CI 0.701-1.473, *P*=.93) for all adverse events. The total number of cardiac events was 8 (1.8%), with 5 (2.3%) cardiac events in the IG and 3 (1.4%) in the CG. Therefore, the intervention effect was not statistically significant (Table 3).

In our Cox regression models, the proportional hazards assumption was tested using Schoenfeld residuals. For the model predicting evaluating the effect of the group on recurrence (with group as the sole covariate), the test statistic was *χ*²_1_=1.11 (*P*=.29). In the model for death, the group test statistic was *χ*²_1_=8.24 (*P*<.05). In the model for all adverse events, the group test statistic was *χ*²_1_=12.87 (*P*<.05).

**Table 3:** Cox regression analysis of patients with stroke (N=432^a^) for endpoints between the IG^b^ and the CG^c^.

Variables			Total participants (N=432), n (%)		IG (n=216), n (%)		CG (n=216), n (%)		Nonadjusted variables				Variables adjusted by baseline data (mRS^d^)		
									HR^e^ (95% CI)		*P* value		HR (95% CI)		*P* value
Recurrence (day 1 to 12 months)			48 (11.1)		25 (11.5)		23 (10.6)		0.978 (0.555-1.722)		.94		1.234 (0.693-2.197)		.48
**Secondary endpoints**															
	Death	95 (22.0)		39 (18.1)		56 (25.9)		1.531 (1.017-2.304)		.04		0.818 (0.540-1.238)		.34	
	Cardiac events	8 (1.8)		5 (2.3)		3 (1.4)		—^f^		—		—		—	
	All adverse events^g^	116 (26.9)		54 (25.0)		62 (28.7)		1.216 (0.844-1.752)		.29		1.016 (0.701-1.473)		.93	

^a^Note that of the 95 (22%) patients who died, only 2 (2.1%) were diagnosed with cardiac events and 33 (34.7%) with recurrence as the cause of death.

^b^IG: intervention group.

^c^CG: control group.

^d^mRS: modified Rankin Scale (mRS score was the covariate).

^e^HR: hazard ratio.

^f^Not applicable.

^g^Calculated by adding total recurrence, death, and cardiac events.

The monthly distribution of all causes of recurrence and death in 12 months was calculated and is shown in Multimedia Appendix 2. Nearly half of the cases of recurrence (27/48, 56.3%) and deaths (47/95, 49.5%) occurred within the first month. We also analyzed the distribution of death across different stroke types and mRS groups (Multimedia Appendix 3) and found that recurrence and death were the highest in the hemorrhagic subtype (n=23, 47.9%, and n=48, 50.5%, respectively) and in patients with an mRS score of 4 (n=31, 64.6%, and n=59, 62.1%, respectively).

### Changes in Medication Adherence Over Time

Medication adherence strongly affects the recurrence of stroke. In this study, we compared the medication intake adherence between the IG and the CG at baseline, midline, and endline. We found significant improvement in the IG at midline and endline (*P*<.001 and *P*=.002, respectively). Taking medication “as doctor’s prescription for 6-7 days per week” significantly increased (Table 4).

**Table 4:** Comparison using the Fisher exact test of medication adherence between the IG^a^ and the CG^b^ at each time point.

Time points and groups		Not at all, n (%)	Occasionally, n (%)	Once/week, n (%)	2-3 days/week, n (%)	4-5 days/week, n (%)	6-7days/week, n (%)
**Baseline (*P*=.78)**							
	IG (n=216)	67 (31.0)	54 (25.0)	1 (0.5)	6 (2.8)	23 (10.6)	65 (30.1)
	CG (n=216)	57 (26.4)	71 (32.9)	0 (0.0)	2 (0.9)	25 (11.6)	61 (28.2)
**Midline^c^ (*P*<.001)**							
	IG (n=149)	12 (8.1)	2 (1.3)	0 (0.0)	10 (6.7)	4 (2.7)	121 (81.2)
	CG (n=130)	26 (20.0)	1 (0.8)	0 (0.0)	0 (0.0)	0 (0.0)	103 (79.2)
**Endline (*P*=.002)**							
	IG (n=166)	2 (1.2)	0 (0.0)	7 (4.2)	5 (3.0)	11 (6.6)	141 (84.9)
	CG (n=141)	11 (7.8)	0 (0.0)	18 (12.8)	5 (3.5)	6 (4.3)	101 (71.6)

^a^IG: intervention group.

^b^CG: control group.

^c^Patient attendance was low at midline.

## Discussion

### Principal Findings

This is the first intervention study that followed patients with stroke for 12 months to evaluate the risk factors for recurrence and other adverse events related to stroke in Bangladesh. We provided health education for controlling modifiable risk factors to prevent the recurrence of stroke in patients who had a stroke attack.

We compared patients who received the health education intervention with those who received usual care, and no significant difference was found in reducing the recurrence of stroke in the IG after 12 months of follow-up. A similar finding was observed in another study: recurrence did not seem to be reduced by only self-management education with treatment coordination [12]. We included disproportionately more patients with severe stroke (mRS score=4) in the CG; therefore, more patients died in the CG. Among all deaths, 61.1% were undiagnosed: 27.4% in the IG and 33.7% in the CG. This caused our intervention to be ineffective.

Patients with stroke need to undergo laboratory investigations, such as computerized tomography (CT) scans and magnetic resonance imaging (MRI) for their diagnosis. However, most of the patients in this study could not undergo these tests for a subsequent attack, recurrence, and other adverse events, including the cause of death, due to the unavailability of investigation facilities and the patients’ financial constraints. Therefore, it was not possible to diagnose recurrence and the cause of death in patients with stroke. In addition, many of our patients could not undergo these tests as they died at home before visiting the hospital for their diagnosis; those deaths were considered of having unknown causes. Among patients with recurrent stroke, less than three-quarters (33/48; n=13 in the IG vs n=20 in the CG) died. Therefore, recurrence was considered a main risk factor for death [21].

We included patients with ischemic and hemorrhagic stroke. The risk of recurrence and death for patients with hemorrhagic stroke is higher compared to those with ischemic stroke [22]. This caused an overall 22% rate of death among the study participants, which might have had a significant impact on the results. Moreover, we did not consider the differences in risk factors, admission severity, and discharge status between the 2 groups, which was also one reason for the insignificant results of this study. It was also difficult to evaluate the effects of the intervention within this short time. Many patients were first diagnosed with new comorbidities, such as hypertension, diabetes, and dyslipidemia, after they visited the hospital and started treatment. Therefore, the risk of recurrence after a stroke remained and made the patients more vulnerable to other adverse events. It also increased the risk of stroke attack after discharge from the hospital due to inadequate monitoring of the risk factors at home. Although the patients were on antihyperlipidemic medication for dyslipidemia, there was still a high risk of developing stroke recurrence [23]. These might have acted as confounding factors that could not reduce the recurrence of stroke significantly through the health education intervention.

The risk of recurrence in our study was higher within the first 6 months compared to the subsequent 6 months. Another study also found that the first 3 months are crucial for the recurrence of stroke [8]. Therefore, intensive intervention is necessary to control the risk factors within this time.

Even though we failed to show the effectiveness of health education in this study, we still cannot ignore the importance of health education. Many previous studies have shown the effectiveness of health education on hypertension, diabetes, and ischemic heart diseases [24-27]. In Bangladesh, postdischarge health education is not paid as much attention in hospitals and primary care settings. Therefore, stroke and other noncommunicable diseases are highly prevalent as a result of this reactive strategy. Nationwide health education programs, routine health checkups, and regular follow-up visits could be preventive measures for the early detection and treatment of the risk factors of stroke. This can also lower the health care costs and enhance long-term health results. Regular health education and health check-up campaigns can increase knowledge about the risk factors of stroke and motivate people to change their behavior. In Bangladesh, patients with stroke attacks usually attend local health care facilities. As the primary and secondary health care systems are weak, patients are not diagnosed properly and treated (lack of qualified physicians and diagnostic facilities) and miss an opportunity to receive acute care management. In our study, patients visited the hospital 7.75 days after the onset of stroke on average, and the IG delayed visiting the hospital more compared to the CG. This higher delay made the IG more vulnerable to developing recurrence and other stroke-related adverse events compared to the CG.

The NINS&H is the only specialized referral hospital in Bangladesh and receives more patients compared to its limited beds. Therefore, only patients with severe stroke (mRS score=3-4) visit the hospital and are admitted, which pushed us to enroll participants from the outpatient department to obtain mRS scores of 0-2.

The sociodemographic attributes of the participants may also be a factor in determining the results. The average age of our participants was 55 years, with a minimum of 18 years to a maximum of 92 years, and males were predominant. One meta-analysis conducted in Japan reported that the average age of the patients was 67.2 years [28]. Considering their age, older adults feel inconvenienced when attending health care facilities due to the long distance from their homes. Despite the desire and necessity of many participants to visit hospitals, financial constraints prevent them from covering treatment-related associated expenses as there is no national health insurance system in Bangladesh [29,30].

It is alarming that young people are also at risk of stroke in this population. Some of them were vulnerable to stroke as they had modifiable risk factors, such as smoking [31,32]. Three-quarters of them had hypertension, and less than three-quarters had dyslipidemia, which are the major risk factors for the recurrence of stroke [23,33]. This study found that medication adherence significantly improved in the IG during monthly follow-ups. This could be a positive effect of health education. Regular medication adherence is crucial for controlling underlying diseases.

About half of our participants were unemployed, and some of them had financial constraints. Their average monthly income was far below (US $ 184.39) the national average income of US $ 277 [34]. Therefore, they might have limited access to hospitals or any health care facilities for medical checkups.

### Limitations

The study has a few limitations. We followed a convenience sampling method using a specialized tertiary hospital that receives patients from diverse geographic regions across the country. Although this approach may have introduced selection bias and limited the representativeness of the sample, we believe the findings provide valuable insights and may still reflect broader patterns relevant to the nationwide setting.

During enrollment, we did not allocate participants to the 2 groups based on the severity of stroke (mRS score); usually, the stroke subtype and the mRS score are correlated [16]. Therefore, there was an uneven distribution of participants, which influenced the findings of our study.

A huge number of participants died at their homes without any investigation for the diagnosis of the cause of death. The limited diagnostic facilities, a lack of health care resources, and patients’ economic difficulties influenced our study findings. The overall sample size was small, and the follow-up time was insufficient, which might also have a significant impact on the results.

Moreover, the number of recurrence cases and the number of events in this study were small, which may have limited the statistical power and widened the CIs, and effects were cautiously interpreted. However, the small number of events and visual inspection suggested time-related potential instability. Causal interpretation should be avoided, and further studies with larger samples and longer follow-ups are needed.

As this study was conducted in the context of Bangladesh, generalizability may be limited due to differences in health care infrastructure, socioeconomic factors, and stroke management practices compared to other countries. We acknowledge that some results, particularly those related to lifestyle behaviors, medication adherence, and self-monitoring, were based on self-reports from patients and caregivers. Although clear guidance was provided to the RA team to ensure accuracy, self-reported data are characteristically topic to social attractiveness and recall biases, which may affect the dependability of the answers. Multicenter studies are recommended to validate the effect of health education for patients poststroke.

This study used a single-blind design, which may have introduced observer bias during outcome assessment, particularly in a resource-limited setting, such as Bangladesh, where familiarity between participants and assessors could influence data collection. To reduce the possibility of assessment bias, outcome evaluations, such as recurrence and medication adherence evaluations, were carried out by an experienced investigator who was not involved in the study.

### Conclusion

We provided health education to patients with stroke and found it had no effect in reducing recurrence and stroke-related adverse events, including death. This study emphasizes the importance of health education in enhancing medication adherence among patients. In both groups, patient with hemorrhagic stroke and an mRS score of 4 had more stroke recurrences and deaths overall. Therefore, the acute care system needs to pay more attention to prevent recurrences and deaths. Patients should visit the hospital without any delay to get treatment in their acute phase. We could not identify the cause of death and recurrence among patients who had not attended health care facilities for their diagnosis and treatment. Introducing regular health checkup systems, postdischarge health education, and follow-up should be incorporated into the national health policy**.** Further interventional studies with large sample sizes to address all limitations, including diagnostic facilities for stroke, will determine the underlying factors for patients with stroke developing recurrence and even dying.

## Appendix

Multimedia Appendix 1 CONSORT 2010 checklist.

Multimedia Appendix 2 Comparison of monthly recurrence and death that occurred during the 6- and 12-month follow-up.

Multimedia Appendix 3 Distribution of recurrence and death due to stroke by type and mRS (modified Rankin Scale) score within the 12-month follow-up period.

## Abbreviations

BP: blood pressure
CG: control group
CONSORT: Consolidated Standards of Reporting Trials
HR: hazard ratio
IG: intervention group
LMIC: low- and middle-income country
mRS: modified Rankin Scale
NINS&H: National Institute of Neuroscience & Hospital
RA: research assistant
SVO: small vessel occlusion
TIA: transient ischemic attack

## Data Availability

The data supporting the findings of this study are available from the corresponding author upon reasonable request.
