# Supplementary material for: Effectiveness of a Health Education Program to Reduce Recurrence of Stroke by Controlling Modifiable Risk Factors in a Specialized Hospital in Bangladesh: Randomized Controlled Trial
Source: JMIR Public Health Surveill. 2025 May 27;11:e72233. doi: 10.2196/72233 (PMC12152434; doi:10.2196/72233)
Supplement: Multimedia Appendix 2 [file publichealth_v11i1e72233_app2.docx]

**Table S1.** Compared the monthly recurrence and death that occurred during the 6 and 12-month follow-up

| **Months of recurrence** | **Total n=432**  **(%)** | **Intervention group**  **n = 216 (%)** | **Control group**  **n = 216 (%)** | ***P*-value** |
| --- | --- | --- | --- | --- |
| Month 1* | 27 (6.3) | 12 (5.6) | 15 (6.9) | 0.551 |
| Month 2 | 2 (0.5) | 0 | 2 (0.9) | 0.156 |
| Month 3 | 5 (1.2) | 3 (1.4) | 2 (0.9) | 0.653 |
| Month 4 | 1 (0.2) | 0 | 1 (0.5) | 0.317 |
| Month 5 | 2 (0.5) | 1 (0.5) | 1 (0.5) | 1.000 |
| Month 6 | 3 (0.7) | 3 (1.4) | 0 |  |
| **Total within 6 months** | 40 (9.2) | 19 (8.8) | 21 (9.7) | 0.740 |
| Month 7 | 3 (0.7) | 3 (1.4) | 0 | 0.82 |
| Month 8 | 1(0.2) | 1(0.5) | 0 | 0.317 |
| Month 9 | 1(0.2) | 0 | 1 (0.5) | 0.317 |
| Month 10 | 0 | 0 |  |  |
| Month 11 | 3 (0.6) | 2 (0.9) | 1(0.5) | 0.562 |
| Month 12 | 0 | 0 | 0 |  |
| **7 to 12 months** | 8 (1.8) | 6 (1.4) | 2 (0.9) | 0.153 |
| **Total after 12 months** | 48 (11.1) | 25 (11.6) | 23 (10.6) | 0.759 |
|  | | | | |
| **Months of all causes of death** | | | | |
| Month 1 | 47 (10.8) | 15 (6.9) | 32 (14.8) | 0.009 |
| Month 2 | 14 (3.2) | 6 (2.8) | 8 (3.7) | 0.587 |
| Month 3 | 6 (1.4) | 2 (0.9) | 4 (1.9) | 0.411 |
| Month 4 | 7 (1.6) | 3 (1.4) | 4 (1.9) | 0.703 |
| Month 5 | 4 (0.9) | 3 (1.4) | 1 (0.5) | 0.315 |
| Month 6 | 4 (0.9) | 3 (1.4) | 1 (0.5) | 0.315 |
| **Total within 6 months** | 82 (19.0%) | 32 (14.1%) | 50 (23.1%) | 0.027 |
| Month 7 | 2 (0.5) | 1 (0.5) | 1 (0.5) | 1.000 |
| Month 8 | 2(0.5) | 1 (0.5) | 1 (0.5) | 1.000 |
| Month 9 | 1 (0.2) | 1 (0.5) | 0 | 0.317 |
| Month 10 | 2 (0.5) | 0 | 2 (0.9) | 0.156 |
| Month 11 | 3 (0.7) | 3 (1.4) | 0 | 0.82 |
| Month 12 | 3 (0.7) | 1 (0.5) | 2 (0.9) | 0.562 |
| **After 7 to 12 months** | 13 (3.01) | 7 (3.2) | 6 (2.8) | 0.778 |
| **Total after 12 months** | 95 (21.9) | 39 (18.0) | 56 (25.9) | 0.048 |

Chi-Square Test, *Month 1 considered 30 days (1-28 days: 26 recurrences, and 29 days: 1 recurrence)
