# Supplementary material for: Effectiveness of a Health Education Program to Reduce Recurrence of Stroke by Controlling Modifiable Risk Factors in a Specialized Hospital in Bangladesh: Randomized Controlled Trial
Source: JMIR Public Health Surveill. 2025 May 27;11:e72233. doi: 10.2196/72233 (PMC12152434; doi:10.2196/72233)
Supplement: Multimedia Appendix 3 [file publichealth_v11i1e72233_app3.docx]

**Table S2.** Distribution of recurrence and death of stroke by types and mRS within 12-month follow-up period

| **Types of stroke** | **Recurrence (n=48)** | **Death (n=95)** |
| --- | --- | --- |
| Cardioembolic | 11(22.9%) | 26 (27.4%) |
| Atherothrombotic | 14 (29.2%) | 20 (21.1%) |
| Lacunar | 0 | 1 (1.1%) |
| Hemorrhagic | 23 (47.9%) | 48 (50.5%) |
| Transient ischemic attack | 0 | 0 |
| **mRS** | **Recurrence (n=48)** | **Death (n=95)** |
| mRS 0 | 0 | 0 |
| mRS 1 | 3 (6.3%) | 2 (2.1%) |
| mRS 2 | 2 (4.2%) | 4 (4.2%) |
| mRS 3 | 12 (25.0%) | 30 (31.6%) |
| mRS 4 | 31 (64.6%) | 59 (62.1%) |
